# Evaluation of a Short-Form of the Berg Card Sorting Test

**DOI:** 10.1371/journal.pone.0063885

**Published:** 2013-05-14

**Authors:** Christopher J. Fox, Shane T. Mueller, Hilary M. Gray, Jacob Raber, Brian J. Piper

**Affiliations:** 1 Department of Behavioral Neuroscience, Oregon Health and Science University, Portland, Oregon, United States of America; 2 Department of Psychology, Portland State University, Portland, Oregon, United States of America; 3 Department of Cognitive and Learning Sciences, Michigan Technological University, Houghton, Michigan, United States of America; 4 Department of Counselor Education, Portland State University, Portland, Oregon, United States of America; 5 Division of Neuroscience, Department of Neurology, ONPRC, Oregon Health & Science University, Portland, Oregon, United States of America; 6 Department of Basic Pharmaceutical Sciences, Husson University, Bangor, Maine, United States of America; University of São Paulo, Brazil

## Abstract

The Psychology Experimental Building Language http://pebl.sourceforge.net/ Berg Card Sorting Test is an open-source neurobehavioral test. Participants (N = 207, ages 6 to 74) completed the Berg Card Sorting Test. Performance on the first 64 trials were isolated and compared to that on the full-length (128 trials) test. Strong correlations between the short and long forms (total errors: r = .87, perseverative response: r = .83, perseverative errors r = .77, categories completed r = .86) support the Berg Card Sorting Test-64 as an abbreviated alternative for the full-length executive function test.

## Introduction

The Berg Card Sorting Test (BCST) is one of many free, non-proprietary neurobehavioral tests provided through Psychology Experiment Building Language (PEBL). PEBL is a software package that allows the creation of computerized tests for experimental use and neuropsychological testing [1], [2]. Like the widely used Wisconsin Card Sorting Test (WCST), the standard version of the BCST contains 128 screen images (cards) and is administered based on procedures described by Berg [3].

Berg's procedures measure executive function and can be useful to identify impairment due to brain disorders or damage. Impaired mental flexibility and diminished executive function can be accurately identified by assessing results of various response categories including total errors, perseverative responses, perseverative errors, and the number of categories completed [4], [5]. Multiple studies have compared the full length and abbreviated versions of the WCST [6]–[9], but similar studies have yet to be performed with the BCST. This investigation explores the possibility of a shorter 64 trial version of the BCST, comparing equality of results with the standard 128 card BCST. It was hypothesized that, based on a data sample from a normative population, the short 64 trial BCST will be as efficient at predicting executive-function capacity as the long 128 trial version.

## Methods

### Participants

Visitors at a science museum in Portland, Oregon, were recruited to participate in this investigation. A total of 207 participants completed testing (95 females and 112 males) and ranged in age from 6 to 74 (*M* = 24.2, *SD* = 16.8).

### Procedure

**Figure 1:**
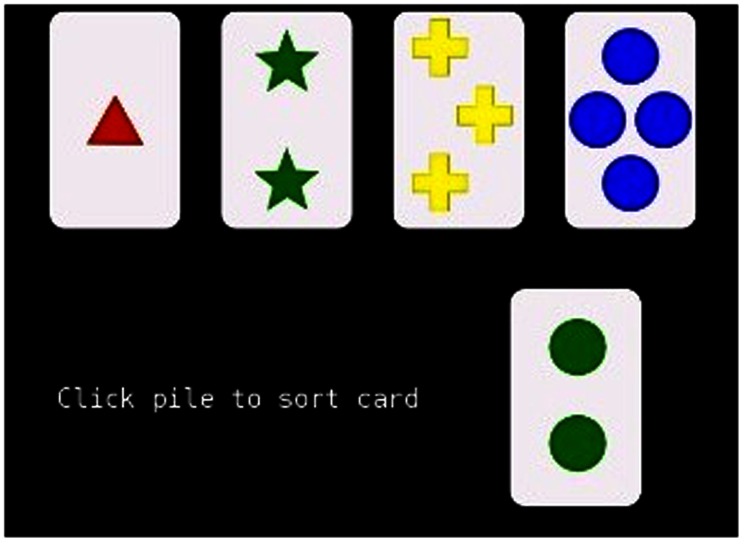
Screen shot showing stimuli from the Psychology Experiment Building Language, version 0.11, (http://pebl.sourceforge.net/) Berg Card Sorting Test.

The standard BCST consists of a 128 card deck displayed on a computer screen. Each card contains a different combination of one of four shapes, colors, and quantities (Figure 1). Four key cards are displayed at the top of the screen as a guide to help determine which of the four stacks the deck's up-card is sorted to. The deck is revealed one card at a time, and the visible card is matched to key cards depending on the particular rule (unknown to the examinee) for a given set. After ten cards have been successfully matched, the set is completed and the sorting rule changes (also unknown to the examinee). The new rule must be discovered using trial and error via feedback received after each card is sorted. After a card is sorted, the participant is provided with feedback regarding whether it was sorted correctly (i.e., according to the current rule). This process continues until the participant either sorts all 128 cards, or until the participant successfully completes 9 sets, whichever comes first. The test can theoretically be completed in as little as 90 trials. However, this is highly unlikely as the participant does not know explicitly when the rule changes or what sort criterion change will occur.

Ten personal computers, used to administrate PEBL 0.10 were placed in a low traffic area of the museum to minimize noise distraction. The participant read the BCST instructions below on the computer screen while simultaneously listening to a researcher who read the instructions aloud.

You are about to take part in an experiment in which you need to categorize cards based on the pictures appearing on them. To begin, you will see four piles (press the mouse button to see the four piles). Each pile has a different number, color, and shape. You will see a series of cards and need to determine which pile each belongs to. Click on a pile with your mouse pointer to determine the pile each new card belongs in. The correct answer depends upon a rule, but you will not know what the rule is. But, we will tell you on each trial whether or not you were correct. Press the mouse button to continue. Finally, the rule may change during the task, so when it does, you should figure out what the rule is as quickly as possible and change with it. Click the mouse button to begin.

Visual feedback stating “correct” or “wrong” was provided following each trial.

Selection of the starting set (color, number, or form) was randomly determined by the software. Additional information regarding the instructions, procedure and sample may be found elsewhere ([10] or http://www.youtube.com/watch?v=iJ9MAuDcFgA ) and the BCST programming code may be found in the supplemental materials. The only interaction allowed during the test was to restate the basic card sorting principal originally given during the instructions, which is consistent with common practice [11]. Verbal consent was provided by each participant or, in the case of minors, by their parents. This strategy was chosen to maximize the confidentiality of the participants. Only after oral consent was obtained would investigators obtain demographic information. All procedures including the consent were approved by the IRB of Oregon Health and Science University (Protocol #03789).

### Data Analysis

SYSTAT (Chicago, IL) version 13.0 was used to perform statistical analysis. Pearson's correlation was used for analyzing all associations. To examine the equality of the full-length and abbreviated versions of the BCST, performance on the first 64 trials were isolated for analysis. Many participants did not require all 128 trials to complete the test, with some finishing in as few as 106 trials ( *M* = 126.5, *SD* = 4.3). Correlations of the percentages of total errors, perseverative responses, perseverative errors, and categories completed within the first 64 trials and the remaining trials were also assessed. The mean and standard deviations were also calculated for these measures. Values were computed based on calculations reported by the PEBL software. It should be noted that the PEBL (Berg) criterion for perseverative responses is considerably more liberal than those outlined in the WCST administration manual (see Table S1 for an example). Nevertheless, some recently published studies have found the PEBL perseverative error measure is sensitive to acute alcohol administration [12], age [10], and, possibly, brain injury [13].

## Results

**Figure 2:**
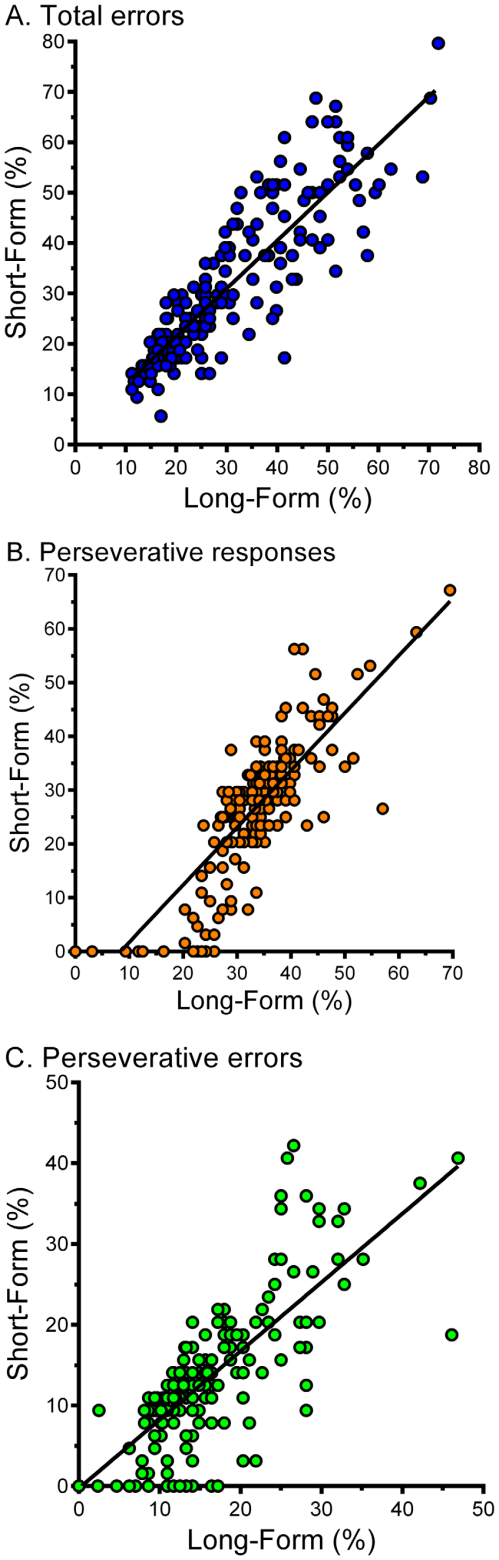
Scatterplots depicting the correlations between percent total error (*r* = 0.87), percent perseverative responses (*r* = 0.83), and percent perseverative errors (*r* = 0.77) among participants (*N* = 207) completing the Psychology Experiment Building Language Berg Card Sorting Test.

Table 1 shows performance on the full-length BCST and for each half separately. The short and long versions of the BCST correlate strongly for percent total errors (*r*(205) = 0.87, *p*<.001, Figure 2A), percent perseverative responses (*r*(205) = 0.83, *p*<.001, Figure 2B), percent perseverative errors (*r*(205) = 0.77, *p*<.001, Figure 2C), and the number of categories completed (*r*(205) = 0.86, *p*<.001). No appreciable difference was found when correlations of females (*r*(93) = 0.80–.89, *p*<0.001) and males (*r*(110) = 0.74–.84, *p*<0.001) were separately examined. Similarly, when correlations were analyzed based on age, a very similar pattern was found for younger and older participants (ages 6–21: *r*(125) = 0.74–.83, *p*<.001, ages 22–74: *r*(78) = 0.81–.91, *p*<0.001).

**Table 1:** Total error, perseverative responses, and perseverative errors among participants (*N* = 207) completing the Psychology Experiment Building Language Berg Card Sorting Test.

	Full-length		Short-form (1^st^ half)		Short-form (2^nd^ half)	
	Raw	%	Raw	%	Raw	%
Total Errors	37.9 (17.6)	29.7 (13.6)	19.7 (9.5)	30.8 (14.8)	18.2 (10.5)	28.7 (16.1)
Persev. Responses	41.1 (13.3)	32.5 (10.3)	16.4 (8.5)	25.7 (13.3)	24.6 (8.1)	39.4 (12.2)
Persev. Errors	19.7 (10.0)	15.5 (7.8)	8.2 (5.5)	12.9 (8.7)	11.4 (6.8)	18.1 (10.5)

Mean and standard deviations are expressed as the raw score and as a percentage of the number of trials completed. Persev  =  Perseverative.

Correlations between performance on the first 64 and the remaining trials were as follows: percent total errors (*r*(205) = 0.55, *p*<0.001), percent perseverative responses (*r*(205) = 0.31, *p*<0.001), percent perseverative errors (*r*(205) = 0.31, *p*<0.001), and categories completed (*r*(205) = 0.51, *p*<.001).

## Discussion

The strong correlations found in this study between performance on the 128-trial and 64-trial versions of the BCST for total errors, perseverative responses, and perseverative errors suggest that the shorter 64-trial BCST is an acceptable alternative to the longer, full-length test. This conclusion is further bolstered when considering the high correlations obtained for subgroups defined by age and sex. These findings are generally concordant with what has been obtained previously with the Wisconsin Card Sorting Test [6]–[9]. Besides cost, a more important advantage of the BCST over the Wisconsin Card Sorting Test is that the Psychology Experiment Building Language tests [1], [2] are open-source which allows for greater openness in how complicated operations are conducted. This transparency is crucial as the Wisconsin Card Sorting Test is the most popular instrument among clinical neuropsychologists for assessing executive function [14] and is considered the gold standard [15].

The correlations between performance on the first 64 trials and the remaining trials were also calculated. These correlations were found to be less strong than the 64-versus-128 comparison. As participants experience the BCST, they learn how the test works, and increasingly become better at identifying when rules shift and how to identify the new rule. With each rule change, a participant can learn how to maximize strategies in order to reduce perseverative responses, though their overall pattern of non-perseverative responses should remain similar, as one or two non-perseverative errors are common after a rule change. The pattern of results obtained may indicate that participants were learning the basic mechanics of the test, resulting in lower perseverative responding as the test progresses, while still preserving a consistent pattern of non-perseverative errors encountered after a rule change.

Card sorting tests based on Berg's principles are used to identify diminished mental capacity due to disorder, disease, and dysfunction including, but not limited to, Alzheimer's disease, schizophrenia, and autism. If the short and long versions of the BCST perform equally, a shorter 64-trial version has many potential benefits. The shorter test duration and immediate computer scored results can be a useful tool when performing neuropsychological evaluations of a subject suspected of deteriorated executive function (e.g. Major Neurocognitive Disorder). The short version can save time if assessment must be performed when time constraints require expedited evaluation and can help preserve an examinee's attention by reducing overall duration when administering a battery of tests.

Two limitations of this investigation are noteworthy. First, the sample consisted of volunteers attending a science museum. It is quite possible that these participants are from a higher socioeconomic background than the general population. In addition, although all museum patrons over the age of five were invited to participate, retirement aged men were less likely to complete the study. We suspect that many of these individuals were concerned that the test might reveal some neurocognitive dysfunction. A second concern is with the experimental design. Although many investigations have isolated performance on the first-half of the WCST trials [6]–[9], some have advocated an alternative design that involves each participant completing both the short and long-forms of a test [16]. This design may provide a more conservative estimate of the correlation between these forms and should be a topic of further investigation.

The current study shows that much of what can be assessed in the 128-trial version of the BCST occurs in the first 64 trials. Future research is needed to determine if this pattern of results also applies to clinical populations.

## Supporting Information

Table S1 **Example sequence of cards, visual feedback on each trial, and the cumulative errors, perseverative responses (PR), perseverative errors (PE), and categories completed (CC) on the Psychology Experiment Building Language Berg Card Sorting Test.**
(DOCX)

File S1 **Folder containing the Psychology Experiment Building Language Berg Card Sorting Test code.**
(ZIP)
