# Supplementary material for: Evaluation of a Short-Form of the Berg Card Sorting Test
Source: PLoS One. 2013 May 14;8(5):e63885. doi: 10.1371/journal.pone.0063885 (PMC3653789; doi:10.1371/journal.pone.0063885)
Supplement: Table S1 — Example sequence of cards, visual feedback on each trial, and the cumulative errors, perseverative responses (PR), perseverative errors (PE), and categories completed (CC) on the Psychology Experiment Building Language Berg Card Sorting Test. (DOCX) [file pone.0063885.s001.docx]

**Supplemental Table 1.** Example sequence of cards, visual feedback on each trial, and the cumulative errors, perseverative responses (PR), perseverative errors (PE), and categories completed (CC) on the Psychology Experiment Building Language Berg Card Sorting Test. The four index cards are one red triangle (#1), two green stars (#2), three yellow plusses (#3), or four blue circles (#4). The strategy employed (C: color, S: shape, or N: number) is listed in parentheses after the selection.

_____________________________________________________________________________________

Card # Stimuli Selection Feedback Total Errors PR PE CC

1 1 circle 1 (N) Incorrect 1 0 0 0

2 1 star 1 (N) Incorrect 2 0 0 0

3 1 triangle 1 (NS) Incorrect 3 0 0 0

4 4 circles 4 (NCS) Correct 3 0 0 0

5 1 plus 4 (C) Correct 3 0 0 0

6 2 circles 1 (C) Correct 3 0 0 0

7 4 triangles 2 (C) Correct 3 0 0 0

8 1 circle 4 (CS) Correct 3 0 0 0

9 2 triangles 1 (S) Incorrect 4 0 0 0

10 2 circles 4 (S) Incorrect 5 0 0 0

11 2 stars 1 (C) Correct 5 0 0 0

12 2 circles 1 (C) Correct 5 0 0 0

13 3 circles 2 (C) Correct 5 0 0 0

14 4 triangles 1 (CS) Correct 5 0 0 0

15 2 plusses 3 (CS) Correct 5 0 0 0

16 2 triangles 1 (CS) Correct 5 0 0 0

17 1 triangle 1 (CSN) Correct 5 0 0 0

18 3 stars 2 (CS) Correct 5 0 0 0

19 2 circles 2 (CN) Correct 5 0 0 0

20 3 triangles 2 (C) Correct^T^ 5 0 0 1

21 4 circles 1 (S) <huh> Incorrect 6 1 1 1

22 1 plus 1 (CN) Incorrect 7 2 2 1

23 4 stars 3 (C) Incorrect 8 3 3 1

24 4 stars 3 (C) Incorrect 9 4 4 1

_____________________________________________________________________________________

^t^trial to complete 1^st^ category
