# Supplementary figures and images for: Evaluation of a Short-Form of the Berg Card Sorting Test
Source: PLoS One. 2013 May 14;8(5):e63885. doi: 10.1371/journal.pone.0063885 (PMC3653789; doi:10.1371/journal.pone.0063885)

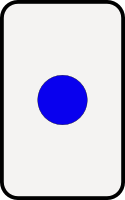

Supplement: File S1 — Folder containing the Psychology Experiment Building Language Berg Card Sorting Test code. (ZIP) [file pone.0063885.s002.zip › bcst_PLOS_One/BCST_Audio64/png/BlueCircle1.png]

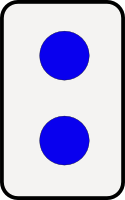

Supplement: File S1 — Folder containing the Psychology Experiment Building Language Berg Card Sorting Test code. (ZIP) [file pone.0063885.s002.zip › bcst_PLOS_One/BCST_Audio64/png/BlueCircle2.png]

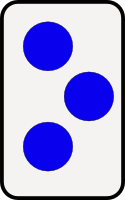

Supplement: File S1 — Folder containing the Psychology Experiment Building Language Berg Card Sorting Test code. (ZIP) [file pone.0063885.s002.zip › bcst_PLOS_One/BCST_Audio64/png/BlueCircle3.png]

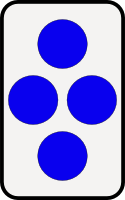

Supplement: File S1 — Folder containing the Psychology Experiment Building Language Berg Card Sorting Test code. (ZIP) [file pone.0063885.s002.zip › bcst_PLOS_One/BCST_Audio64/png/BlueCircle4.png]

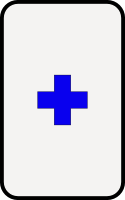

Supplement: File S1 — Folder containing the Psychology Experiment Building Language Berg Card Sorting Test code. (ZIP) [file pone.0063885.s002.zip › bcst_PLOS_One/BCST_Audio64/png/BlueCross1.png]

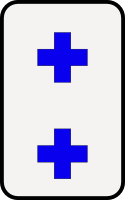

Supplement: File S1 — Folder containing the Psychology Experiment Building Language Berg Card Sorting Test code. (ZIP) [file pone.0063885.s002.zip › bcst_PLOS_One/BCST_Audio64/png/BlueCross2.png]

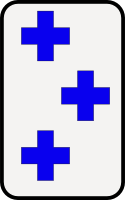

Supplement: File S1 — Folder containing the Psychology Experiment Building Language Berg Card Sorting Test code. (ZIP) [file pone.0063885.s002.zip › bcst_PLOS_One/BCST_Audio64/png/BlueCross3.png]

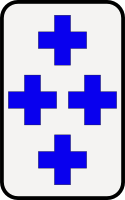

Supplement: File S1 — Folder containing the Psychology Experiment Building Language Berg Card Sorting Test code. (ZIP) [file pone.0063885.s002.zip › bcst_PLOS_One/BCST_Audio64/png/BlueCross4.png]

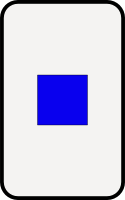

Supplement: File S1 — Folder containing the Psychology Experiment Building Language Berg Card Sorting Test code. (ZIP) [file pone.0063885.s002.zip › bcst_PLOS_One/BCST_Audio64/png/BlueSquare1.png]

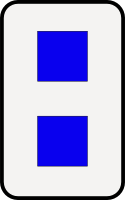

Supplement: File S1 — Folder containing the Psychology Experiment Building Language Berg Card Sorting Test code. (ZIP) [file pone.0063885.s002.zip › bcst_PLOS_One/BCST_Audio64/png/BlueSquare2.png]

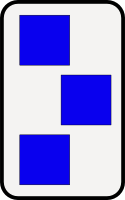

Supplement: File S1 — Folder containing the Psychology Experiment Building Language Berg Card Sorting Test code. (ZIP) [file pone.0063885.s002.zip › bcst_PLOS_One/BCST_Audio64/png/BlueSquare3.png]

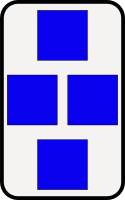

Supplement: File S1 — Folder containing the Psychology Experiment Building Language Berg Card Sorting Test code. (ZIP) [file pone.0063885.s002.zip › bcst_PLOS_One/BCST_Audio64/png/BlueSquare4.png]

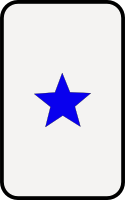

Supplement: File S1 — Folder containing the Psychology Experiment Building Language Berg Card Sorting Test code. (ZIP) [file pone.0063885.s002.zip › bcst_PLOS_One/BCST_Audio64/png/BlueStar1.png]

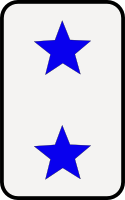

Supplement: File S1 — Folder containing the Psychology Experiment Building Language Berg Card Sorting Test code. (ZIP) [file pone.0063885.s002.zip › bcst_PLOS_One/BCST_Audio64/png/BlueStar2.png]

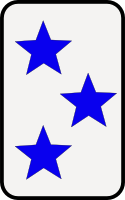

Supplement: File S1 — Folder containing the Psychology Experiment Building Language Berg Card Sorting Test code. (ZIP) [file pone.0063885.s002.zip › bcst_PLOS_One/BCST_Audio64/png/BlueStar3.png]

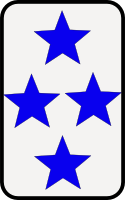

Supplement: File S1 — Folder containing the Psychology Experiment Building Language Berg Card Sorting Test code. (ZIP) [file pone.0063885.s002.zip › bcst_PLOS_One/BCST_Audio64/png/BlueStar4.png]

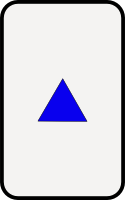

Supplement: File S1 — Folder containing the Psychology Experiment Building Language Berg Card Sorting Test code. (ZIP) [file pone.0063885.s002.zip › bcst_PLOS_One/BCST_Audio64/png/BlueTriangle1.png]

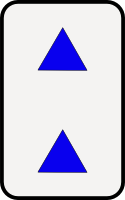

Supplement: File S1 — Folder containing the Psychology Experiment Building Language Berg Card Sorting Test code. (ZIP) [file pone.0063885.s002.zip › bcst_PLOS_One/BCST_Audio64/png/BlueTriangle2.png]

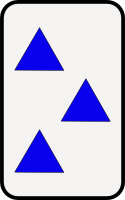

Supplement: File S1 — Folder containing the Psychology Experiment Building Language Berg Card Sorting Test code. (ZIP) [file pone.0063885.s002.zip › bcst_PLOS_One/BCST_Audio64/png/BlueTriangle3.png]

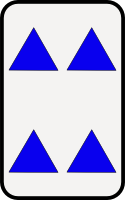

Supplement: File S1 — Folder containing the Psychology Experiment Building Language Berg Card Sorting Test code. (ZIP) [file pone.0063885.s002.zip › bcst_PLOS_One/BCST_Audio64/png/BlueTriangle4.png]

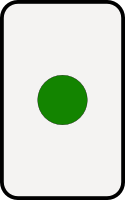

Supplement: File S1 — Folder containing the Psychology Experiment Building Language Berg Card Sorting Test code. (ZIP) [file pone.0063885.s002.zip › bcst_PLOS_One/BCST_Audio64/png/GreenCircle1.png]

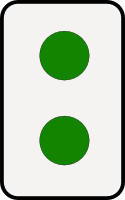

Supplement: File S1 — Folder containing the Psychology Experiment Building Language Berg Card Sorting Test code. (ZIP) [file pone.0063885.s002.zip › bcst_PLOS_One/BCST_Audio64/png/GreenCircle2.png]

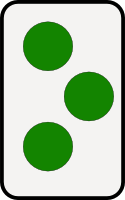

Supplement: File S1 — Folder containing the Psychology Experiment Building Language Berg Card Sorting Test code. (ZIP) [file pone.0063885.s002.zip › bcst_PLOS_One/BCST_Audio64/png/GreenCircle3.png]

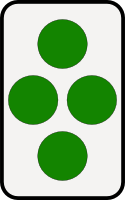

Supplement: File S1 — Folder containing the Psychology Experiment Building Language Berg Card Sorting Test code. (ZIP) [file pone.0063885.s002.zip › bcst_PLOS_One/BCST_Audio64/png/GreenCircle4.png]

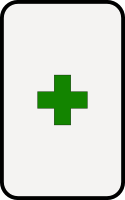

Supplement: File S1 — Folder containing the Psychology Experiment Building Language Berg Card Sorting Test code. (ZIP) [file pone.0063885.s002.zip › bcst_PLOS_One/BCST_Audio64/png/GreenCross1.png]

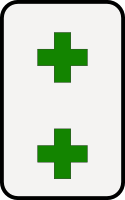

Supplement: File S1 — Folder containing the Psychology Experiment Building Language Berg Card Sorting Test code. (ZIP) [file pone.0063885.s002.zip › bcst_PLOS_One/BCST_Audio64/png/GreenCross2.png]

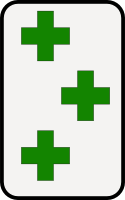

Supplement: File S1 — Folder containing the Psychology Experiment Building Language Berg Card Sorting Test code. (ZIP) [file pone.0063885.s002.zip › bcst_PLOS_One/BCST_Audio64/png/GreenCross3.png]

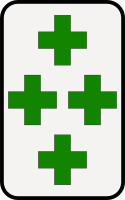

Supplement: File S1 — Folder containing the Psychology Experiment Building Language Berg Card Sorting Test code. (ZIP) [file pone.0063885.s002.zip › bcst_PLOS_One/BCST_Audio64/png/GreenCross4.png]

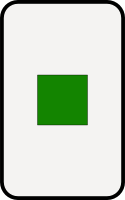

Supplement: File S1 — Folder containing the Psychology Experiment Building Language Berg Card Sorting Test code. (ZIP) [file pone.0063885.s002.zip › bcst_PLOS_One/BCST_Audio64/png/GreenSquare1.png]

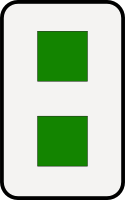

Supplement: File S1 — Folder containing the Psychology Experiment Building Language Berg Card Sorting Test code. (ZIP) [file pone.0063885.s002.zip › bcst_PLOS_One/BCST_Audio64/png/GreenSquare2.png]

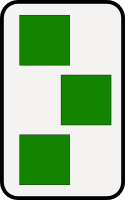

Supplement: File S1 — Folder containing the Psychology Experiment Building Language Berg Card Sorting Test code. (ZIP) [file pone.0063885.s002.zip › bcst_PLOS_One/BCST_Audio64/png/GreenSquare3.png]

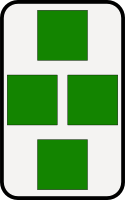

Supplement: File S1 — Folder containing the Psychology Experiment Building Language Berg Card Sorting Test code. (ZIP) [file pone.0063885.s002.zip › bcst_PLOS_One/BCST_Audio64/png/GreenSquare4.png]

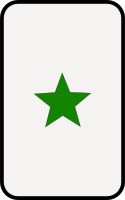

Supplement: File S1 — Folder containing the Psychology Experiment Building Language Berg Card Sorting Test code. (ZIP) [file pone.0063885.s002.zip › bcst_PLOS_One/BCST_Audio64/png/GreenStar1.png]

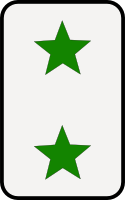

Supplement: File S1 — Folder containing the Psychology Experiment Building Language Berg Card Sorting Test code. (ZIP) [file pone.0063885.s002.zip › bcst_PLOS_One/BCST_Audio64/png/GreenStar2.png]

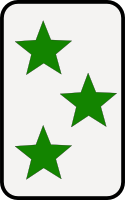

Supplement: File S1 — Folder containing the Psychology Experiment Building Language Berg Card Sorting Test code. (ZIP) [file pone.0063885.s002.zip › bcst_PLOS_One/BCST_Audio64/png/GreenStar3.png]

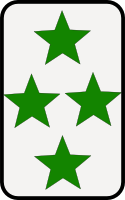

Supplement: File S1 — Folder containing the Psychology Experiment Building Language Berg Card Sorting Test code. (ZIP) [file pone.0063885.s002.zip › bcst_PLOS_One/BCST_Audio64/png/GreenStar4.png]

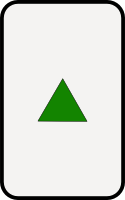

Supplement: File S1 — Folder containing the Psychology Experiment Building Language Berg Card Sorting Test code. (ZIP) [file pone.0063885.s002.zip › bcst_PLOS_One/BCST_Audio64/png/GreenTriangle1.png]

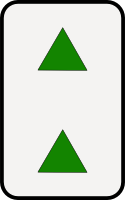

Supplement: File S1 — Folder containing the Psychology Experiment Building Language Berg Card Sorting Test code. (ZIP) [file pone.0063885.s002.zip › bcst_PLOS_One/BCST_Audio64/png/GreenTriangle2.png]

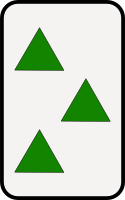

Supplement: File S1 — Folder containing the Psychology Experiment Building Language Berg Card Sorting Test code. (ZIP) [file pone.0063885.s002.zip › bcst_PLOS_One/BCST_Audio64/png/GreenTriangle3.png]

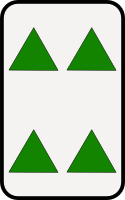

Supplement: File S1 — Folder containing the Psychology Experiment Building Language Berg Card Sorting Test code. (ZIP) [file pone.0063885.s002.zip › bcst_PLOS_One/BCST_Audio64/png/GreenTriangle4.png]

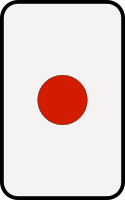

Supplement: File S1 — Folder containing the Psychology Experiment Building Language Berg Card Sorting Test code. (ZIP) [file pone.0063885.s002.zip › bcst_PLOS_One/BCST_Audio64/png/RedCircle1.png]

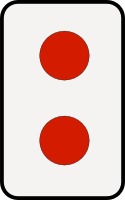

Supplement: File S1 — Folder containing the Psychology Experiment Building Language Berg Card Sorting Test code. (ZIP) [file pone.0063885.s002.zip › bcst_PLOS_One/BCST_Audio64/png/RedCircle2.png]

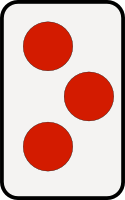

Supplement: File S1 — Folder containing the Psychology Experiment Building Language Berg Card Sorting Test code. (ZIP) [file pone.0063885.s002.zip › bcst_PLOS_One/BCST_Audio64/png/RedCircle3.png]

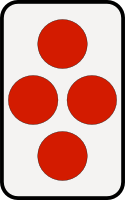

Supplement: File S1 — Folder containing the Psychology Experiment Building Language Berg Card Sorting Test code. (ZIP) [file pone.0063885.s002.zip › bcst_PLOS_One/BCST_Audio64/png/RedCircle4.png]

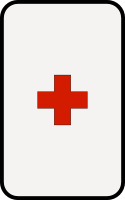

Supplement: File S1 — Folder containing the Psychology Experiment Building Language Berg Card Sorting Test code. (ZIP) [file pone.0063885.s002.zip › bcst_PLOS_One/BCST_Audio64/png/RedCross1.png]

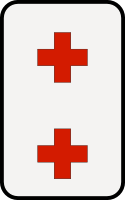

Supplement: File S1 — Folder containing the Psychology Experiment Building Language Berg Card Sorting Test code. (ZIP) [file pone.0063885.s002.zip › bcst_PLOS_One/BCST_Audio64/png/RedCross2.png]

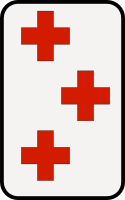

Supplement: File S1 — Folder containing the Psychology Experiment Building Language Berg Card Sorting Test code. (ZIP) [file pone.0063885.s002.zip › bcst_PLOS_One/BCST_Audio64/png/RedCross3.png]

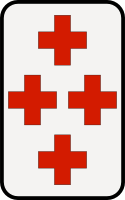

Supplement: File S1 — Folder containing the Psychology Experiment Building Language Berg Card Sorting Test code. (ZIP) [file pone.0063885.s002.zip › bcst_PLOS_One/BCST_Audio64/png/RedCross4.png]

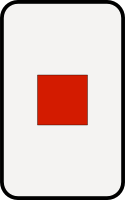

Supplement: File S1 — Folder containing the Psychology Experiment Building Language Berg Card Sorting Test code. (ZIP) [file pone.0063885.s002.zip › bcst_PLOS_One/BCST_Audio64/png/RedSquare1.png]

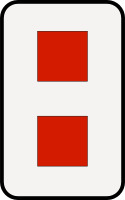

Supplement: File S1 — Folder containing the Psychology Experiment Building Language Berg Card Sorting Test code. (ZIP) [file pone.0063885.s002.zip › bcst_PLOS_One/BCST_Audio64/png/RedSquare2.png]

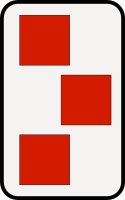

Supplement: File S1 — Folder containing the Psychology Experiment Building Language Berg Card Sorting Test code. (ZIP) [file pone.0063885.s002.zip › bcst_PLOS_One/BCST_Audio64/png/RedSquare3.png]

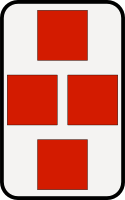

Supplement: File S1 — Folder containing the Psychology Experiment Building Language Berg Card Sorting Test code. (ZIP) [file pone.0063885.s002.zip › bcst_PLOS_One/BCST_Audio64/png/RedSquare4.png]

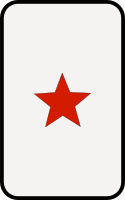

Supplement: File S1 — Folder containing the Psychology Experiment Building Language Berg Card Sorting Test code. (ZIP) [file pone.0063885.s002.zip › bcst_PLOS_One/BCST_Audio64/png/RedStar1.png]

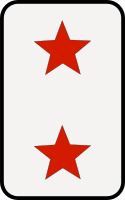

Supplement: File S1 — Folder containing the Psychology Experiment Building Language Berg Card Sorting Test code. (ZIP) [file pone.0063885.s002.zip › bcst_PLOS_One/BCST_Audio64/png/RedStar2.png]

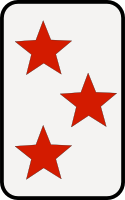

Supplement: File S1 — Folder containing the Psychology Experiment Building Language Berg Card Sorting Test code. (ZIP) [file pone.0063885.s002.zip › bcst_PLOS_One/BCST_Audio64/png/RedStar3.png]

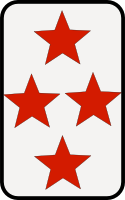

Supplement: File S1 — Folder containing the Psychology Experiment Building Language Berg Card Sorting Test code. (ZIP) [file pone.0063885.s002.zip › bcst_PLOS_One/BCST_Audio64/png/RedStar4.png]

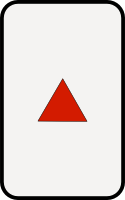

Supplement: File S1 — Folder containing the Psychology Experiment Building Language Berg Card Sorting Test code. (ZIP) [file pone.0063885.s002.zip › bcst_PLOS_One/BCST_Audio64/png/RedTriangle1.png]

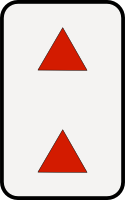

Supplement: File S1 — Folder containing the Psychology Experiment Building Language Berg Card Sorting Test code. (ZIP) [file pone.0063885.s002.zip › bcst_PLOS_One/BCST_Audio64/png/RedTriangle2.png]

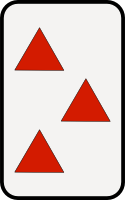

Supplement: File S1 — Folder containing the Psychology Experiment Building Language Berg Card Sorting Test code. (ZIP) [file pone.0063885.s002.zip › bcst_PLOS_One/BCST_Audio64/png/RedTriangle3.png]

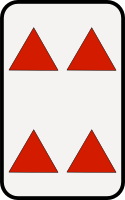

Supplement: File S1 — Folder containing the Psychology Experiment Building Language Berg Card Sorting Test code. (ZIP) [file pone.0063885.s002.zip › bcst_PLOS_One/BCST_Audio64/png/RedTriangle4.png]

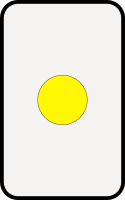

Supplement: File S1 — Folder containing the Psychology Experiment Building Language Berg Card Sorting Test code. (ZIP) [file pone.0063885.s002.zip › bcst_PLOS_One/BCST_Audio64/png/YellowCircle1.png]

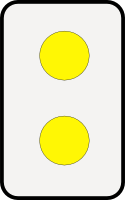

Supplement: File S1 — Folder containing the Psychology Experiment Building Language Berg Card Sorting Test code. (ZIP) [file pone.0063885.s002.zip › bcst_PLOS_One/BCST_Audio64/png/YellowCircle2.png]

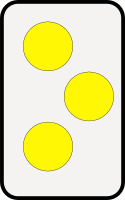

Supplement: File S1 — Folder containing the Psychology Experiment Building Language Berg Card Sorting Test code. (ZIP) [file pone.0063885.s002.zip › bcst_PLOS_One/BCST_Audio64/png/YellowCircle3.png]

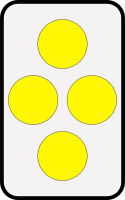

Supplement: File S1 — Folder containing the Psychology Experiment Building Language Berg Card Sorting Test code. (ZIP) [file pone.0063885.s002.zip › bcst_PLOS_One/BCST_Audio64/png/YellowCircle4.png]

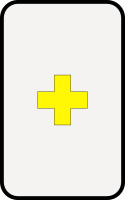

Supplement: File S1 — Folder containing the Psychology Experiment Building Language Berg Card Sorting Test code. (ZIP) [file pone.0063885.s002.zip › bcst_PLOS_One/BCST_Audio64/png/YellowCross1.png]

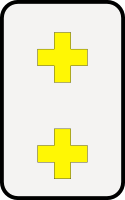

Supplement: File S1 — Folder containing the Psychology Experiment Building Language Berg Card Sorting Test code. (ZIP) [file pone.0063885.s002.zip › bcst_PLOS_One/BCST_Audio64/png/YellowCross2.png]

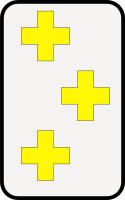

Supplement: File S1 — Folder containing the Psychology Experiment Building Language Berg Card Sorting Test code. (ZIP) [file pone.0063885.s002.zip › bcst_PLOS_One/BCST_Audio64/png/YellowCross3.png]

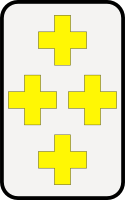

Supplement: File S1 — Folder containing the Psychology Experiment Building Language Berg Card Sorting Test code. (ZIP) [file pone.0063885.s002.zip › bcst_PLOS_One/BCST_Audio64/png/YellowCross4.png]

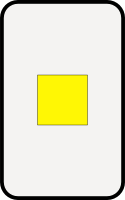

Supplement: File S1 — Folder containing the Psychology Experiment Building Language Berg Card Sorting Test code. (ZIP) [file pone.0063885.s002.zip › bcst_PLOS_One/BCST_Audio64/png/YellowSquare1.png]

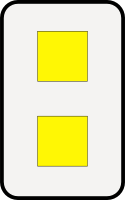

Supplement: File S1 — Folder containing the Psychology Experiment Building Language Berg Card Sorting Test code. (ZIP) [file pone.0063885.s002.zip › bcst_PLOS_One/BCST_Audio64/png/YellowSquare2.png]

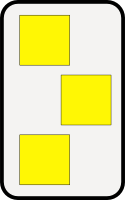

Supplement: File S1 — Folder containing the Psychology Experiment Building Language Berg Card Sorting Test code. (ZIP) [file pone.0063885.s002.zip › bcst_PLOS_One/BCST_Audio64/png/YellowSquare3.png]

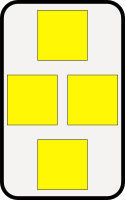

Supplement: File S1 — Folder containing the Psychology Experiment Building Language Berg Card Sorting Test code. (ZIP) [file pone.0063885.s002.zip › bcst_PLOS_One/BCST_Audio64/png/YellowSquare4.png]

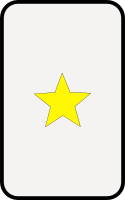

Supplement: File S1 — Folder containing the Psychology Experiment Building Language Berg Card Sorting Test code. (ZIP) [file pone.0063885.s002.zip › bcst_PLOS_One/BCST_Audio64/png/YellowStar1.png]

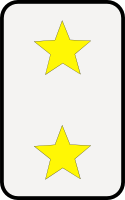

Supplement: File S1 — Folder containing the Psychology Experiment Building Language Berg Card Sorting Test code. (ZIP) [file pone.0063885.s002.zip › bcst_PLOS_One/BCST_Audio64/png/YellowStar2.png]

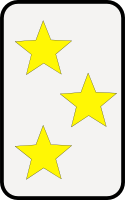

Supplement: File S1 — Folder containing the Psychology Experiment Building Language Berg Card Sorting Test code. (ZIP) [file pone.0063885.s002.zip › bcst_PLOS_One/BCST_Audio64/png/YellowStar3.png]

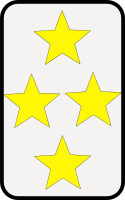

Supplement: File S1 — Folder containing the Psychology Experiment Building Language Berg Card Sorting Test code. (ZIP) [file pone.0063885.s002.zip › bcst_PLOS_One/BCST_Audio64/png/YellowStar4.png]

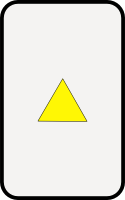

Supplement: File S1 — Folder containing the Psychology Experiment Building Language Berg Card Sorting Test code. (ZIP) [file pone.0063885.s002.zip › bcst_PLOS_One/BCST_Audio64/png/YellowTriangle1.png]

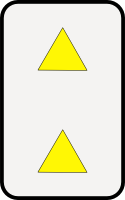

Supplement: File S1 — Folder containing the Psychology Experiment Building Language Berg Card Sorting Test code. (ZIP) [file pone.0063885.s002.zip › bcst_PLOS_One/BCST_Audio64/png/YellowTriangle2.png]

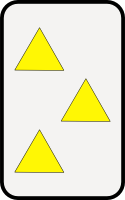

Supplement: File S1 — Folder containing the Psychology Experiment Building Language Berg Card Sorting Test code. (ZIP) [file pone.0063885.s002.zip › bcst_PLOS_One/BCST_Audio64/png/YellowTriangle3.png]

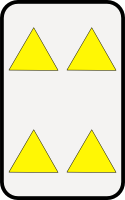

Supplement: File S1 — Folder containing the Psychology Experiment Building Language Berg Card Sorting Test code. (ZIP) [file pone.0063885.s002.zip › bcst_PLOS_One/BCST_Audio64/png/YellowTriangle4.png]
